# Diabetes mellitus activates fetal gene program and intensifies cardiac remodeling and oxidative stress in aged spontaneously hypertensive rats

**DOI:** 10.1186/1475-2840-12-152

**Published:** 2013-10-17

**Authors:** Camila Moreno Rosa, Natasha Priscilla Xavier, Dijon Henrique Campos, Ana Angélica Henrique Fernandes, Marcelo Diarcadia Mariano Cezar, Paula Felippe Martinez, Antonio Carlos Cicogna, Camila Gimenes, Rodrigo Gimenes, Marina Politi Okoshi, Katashi Okoshi

**Affiliations:** 1 Department of Internal Medicine, Botucatu Medical School, Sao Paulo State University, UNESP, Botucatu, Brazil; 2 Department of Chemistry and Biochemistry, Institute of Biosciences, Sao Paulo State University, UNESP, Botucatu, Brazil; 3 Departamento de Clinica Medica, Faculdade de Medicina de Botucatu, UNESP, Rubiao Junior, S/N 18618-970, Botucatu, SP, Brazil

**Keywords:** Diabetes mellitus, Oxidative stress, Systemic hypertension, Spontaneously hypertensive rats, Elderly

## Abstract

**Background:**

The combination of systemic arterial hypertension and diabetes mellitus (DM) induces greater cardiac remodeling than either condition alone. However, this association has been poorly addressed in senescent rats. Therefore, this study aimed to analyze the influence of streptozotocin-induced DM on ventricular remodeling and oxidative stress in aged spontaneously hypertensive rats (SHR)**.**

**Methods:**

Fifty 18 month old male SHR were divided into two groups: control (SHR, n = 25) and diabetic (SHR-DM, n = 25). DM was induced by streptozotocin (40 mg/kg, i.p.). After nine weeks, the rats underwent echocardiography and myocardial functional study in left ventricular (LV) isolated papillary muscle preparations. LV samples were obtained to measure myocyte diameters, interstitial collagen fraction, and hydroxyproline concentration. Gene expression of atrial natriuretic peptide (ANP) and α- and β-myosin heavy chain (MyHC) isoforms was evaluated by RT-PCR. Serum oxidative stress was assessed by measuring lipid hydroperoxide concentration and superoxide dismutase and glutathione peroxidase activities. Statistics: Student’s *t* test or Mann-Whitney test, p < 0.05.

**Results:**

SHR-DM presented higher blood glucose (487 ± 29 vs. 89.1 ± 21.1 mg/dL) and lower body weight (277 ± 26 vs. 339 ± 38 g). Systolic blood pressure did not differ between groups. Echocardiography showed LV and left atrial dilation, LV diastolic and relative wall thickness decrease, and LV systolic and diastolic function impairment in SHR-DM. Papillary muscle study showed decreased myocardial contractility and contractile reserve in SHR-DM. Myocyte diameters and myocardial interstitial collagen fraction and hydroxyproline concentration did not differ between groups. Increased serum pro-oxidant activity and gene expression of ANP and β/α-MyHC ratio were observed in DM.

**Conclusion:**

Diabetes mellitus induces cardiac dilation and functional impairment, increases oxidative stress and activates fetal gene program in aged spontaneously hypertensive rats.

## Background

Heart failure represents a major public health issue reaching epidemic proportions especially in elderly populations [1]. The prevalence of risk factors for cardiac failure is increasing at an alarming rate. Diabetes mellitus (DM) and systemic arterial hypertension, important predisposing factors, are associated with increased morbidity and mortality [1].

Diabetes mellitus is a major risk factor for the development of coronary atherosclerosis and diabetic cardiomyopathy, a non-atherogenic myocardial disease. Diabetic cardiomyopathy results from complex relationships between metabolic abnormalities that accompany diabetes and their cellular consequences leading to alterations in myocardial structure and function [2-5]. Diabetes induced cardiac remodeling is characterized by myocardial interstitial and perivascular fibrosis, myocyte hypertrophy, calcium handling and contractile proteins abnormalities, and abnormal cardiomyocytes loss by apoptosis [2,6,7]. Hyperglycemia induced oxidative stress is an important pathological mechanism involved in diabetic cardiomyopathy [2,8].

Diabetes in humans is often associated with systemic arterial hypertension. The combination of DM and hypertension induces greater structural and functional cardiac damage than either condition alone [9]. This condition has been called diabetic hypertensive cardiomyopathy [2]. However, the physiopathology of diabetes and hypertension induced cardiac injury is not completely understood. In experimental studies, diabetes in hypertensive rodents has very often resulted in impaired cardiac remodeling by exacerbating fibrosis, changing pro-hypertrophic factors expression, and inducing inflammation, microvascular changes, and higher mortality rate than isolated DM or hypertension [10-16]**.** Although the prevalence of risk factors for cardiovascular disease increases with age, the combined effects of diabetes and hypertension on cardiac remodeling during senescence have been poorly addressed.

One widely used experimental model for studying hypertension and heart failure is the spontaneously hypertensive rat (SHR) [17-19]. It presents early arterial hypertension and left ventricular hypertrophy [20] which evolves to heart failure during maturity and senescence. As cardiac failure development is slow, SHRs are considered a useful model for mimicking clinical heart failure. The aim of this study was to analyze the influence of streptozotocin-induced DM on cardiac remodeling and oxidative stress in aged SHR.

## Methods

### Experimental groups

Male SHR were obtained from the Central Animal House at Botucatu Medical School, UNESP. All animals were housed in a room under temperature control at 23°C and kept on a 12-hour light/dark cycle. Food and water were supplied *ad libitum*. All experiments and procedures were approved by the Ethics Committee of Botucatu Medical School, UNESP, Botucatu, SP, Brazil.

Eighteen-month-old SHR were assigned into two groups: control (SHR, n = 25) and diabetic (SHR-DM, n = 25). Diabetes was induced by intraperitoneal injection of streptozotocin (Sigma, St. Louis, MO, USA) at 40 mg/kg body weight diluted in 0.01 M citrate buffer pH 4.5 [21]. The control group received intraperitoneal injection of vehicle only. Seven days after streptozotocin administration, blood glucose was measured by glucometer (Advantage®). Only streptozotocin rats with glycemia >220 mg/dL were considered diabetic and included in the study [22]. Rats were studied nine weeks after streptozotocin administration. Previous studies in normotensive rats by our laboratory [23] and other authors [4] have shown dilated left ventricle and impaired systolic function 8-9 weeks after streptozotocin injection.

Systolic arterial pressure was measured before streptozotocin injection and at the end of experiment by tail-cuff method using a model 709-0610 electro-sphygmomanometer (*Narco Bio-System*®, *International Biomedical Inc*., USA).

### Echocardiographic study

Echocardiographic evaluation was performed in all rats using a commercially available echocardiograph (General Electric Medical Systems, Vivid S6, Tirat Carmel, Israel) equipped with a 5-11.5 MHz multifrequency probe, as previously described [19,24,25]. Rats were anesthetized by intramuscular injection of a mixture of ketamine (50 mg/kg) and xylazine (0.5 mg/kg). A two-dimensional parasternal short-axis view of the LV was obtained at the level of the papillary muscles. M-mode tracings were obtained from short-axis views of the LV at or just below the tip of the mitral-valve leaflets, and at the level of the aortic valve and left atrium [26]. M-mode images of the LV were printed on a black-and-white thermal printer (Sony UP-890MD) at a sweep speed of 100 mm/s. All LV structures were manually measured by the same observer as previously described [26]. Values obtained were the mean of at least five cardiac cycles on M-mode tracings. The following structural variables were measured: left atrium diameter (LA), LV diastolic and systolic diameters (LVDD and LVSD, respectively), LV diastolic posterior wall thickness (PWT), LV diastolic anterior wall thickness (AWT), and aortic diameter (AO). Left ventricular weight (LVW) was calculated using the formula [(LVDD + PWT + AWT)^3^ – (LVDD)^3^] × 1.04 [27]. Left ventricular function was assessed by the following parameters: endocardial fractional shortening (FS), ejection fraction (EF), posterior wall shortening velocity (PWSV), early and late diastolic mitral inflow velocities (E and A waves), E/A ratio, E-wave deceleration time (EDT), and isovolumetric relaxation time (IVRT). As body weight differed between groups, cardiac structures were presented in both absolute and values normalized to body weight and tibia length (measured during euthanasia).

### Myocardial functional evaluation

At the end of the experimental period, myocardial contractile performance was evaluated in all rats using isolated LV papillary muscle preparation as previously described [28,29]. Rats were anesthetized (pentobarbital sodium, 50 mg/kg, intraperitoneally) and decapitated. Hearts were quickly removed and placed in oxygenated Krebs-Henseleit solution at 28°C. Left ventricular anterior or posterior papillary muscle was dissected free, mounted between two spring clips, and placed vertically in a chamber containing Krebs-Henseleit solution at 28°C and oxygenated with a mixture of 95% O_2_ and 5% CO_2_ (pH 7.38). The composition of the Krebs-Henseleit solution in mM was as follows: 118.5 NaCl, 4.69 KCl, 1.25 CaCl_2_, 1.16 MgSO_4_, 1.18 KH_2_PO_4_, 5.50 glucose, and 25.88 NaHCO_3_. The spring clips were attached to a Kyowa model 120 T-20B force transducer and a lever system, which allowed for muscle length adjustment. Preparations were stimulated 12 times/min at a voltage 10% above threshold.

After a 60-min period, during which the preparations were permitted to shorten while carrying light loads, muscles were loaded to contract isometrically and stretched to the apices of their length-tension curves. After a 5-min period, during which preparations performed isotonic contractions, muscles were again placed under isometric conditions, and the apex of the length-tension curve (L_max_) was determined. A 15 minute period of stable isometric contraction was imposed prior to the experimental period. One isometric contraction was then recorded for later analysis.

The following parameters were measured from the isometric contraction: peak developed tension (DT, g/mm^2^), resting tension (RT, g/mm^2^), time to peak tension (TPT, ms), maximum tension development rate (+dT/dt, g/mm^2^/s), and maximum tension decline rate (-dT/dt, g/mm^2^/s). To evaluate contractile reserve, the mechanical performance of papillary muscles was evaluated at basal condition and after the following inotropic stimulation: post-rest contractions, extracellular Ca^2+^ concentration increase, and beta-adrenergic agonist isoproterenol added to the nutrient solution.

Papillary muscle cross-sectional area (CSA) was calculated from muscle weight and length by assuming cylindrical uniformity and a specific gravity of 1.0. All force data were normalized for muscle CSA. Papillary muscles with CSA >1.7 mm^2^ were excluded from analysis as muscles with CSA >1.7 mm^2^ can present central core hypoxia and impaired functional performance.

### Histologic analysis

Transverse LV sections were fixed in 10% buffered formalin and embedded in paraffin. Sections (5 μm thick) were stained with hematoxylin–eosin and collagen-specific stain picrosirius red (Sirius red F3BA in aqueous saturated picric acid) [30]. In at least 50 myocytes from each LV, where the nucleus could clearly be identified, the smallest transverse diameters were measured [31]. On average, 20 microscopic fields were used to quantify interstitial collagen fraction. Perivascular collagen was excluded from this analysis [30,32]. Measurements were performed using a Leica microscope (magnification 40×) attached to a video camera and connected to a computer equipped with image analysis software (Image-Pro Plus 3.0, Media Cybernetics, Silver Spring, MD, USA).

### Myocardial hydroxyproline concentration

Myocardial hydroxyproline concentration was measured to estimate myocardial collagen content. Hydroxyproline was measured in LV tissue [28,33]. Briefly, tissue was dried using a Speedvac Concentrator (SC 100) attached to a refrigerated condensation trap (TRL 100) and vacuum pump (VP 100, Savant Instruments, Inc., Farmingdale, NY, USA). Tissue dry weight was measured and samples were hydrolyzed overnight at 100°C with 6 N HCl (1 mL/10 mg dry tissue). An 50 μL aliquot of the hydrolysate was transferred to an Eppendorf tube and dried in the Speedvac Concentrator. One milliliter of deionized water was added and the sample transferred to a tube with a Teflon screw cap. One milliliter of potassium borate buffer (pH 8.7) was added to maintain constant pH and the sample was oxidized with 0.3 mL of chloramine T solution at room temperature for 20 minutes. The addition of 1 mL of 3.6 M/L sodium thiosulfate with thorough mixing for 10 s stopped the oxidative process. The solution was saturated with 1.5 g KCl. The tubes were capped and heated in boiling water for 20 min. After cooling to room temperature, the aqueous layer was extracted with 2.5 mL of toluene. One and a half milliliter of toluene extract was transferred to a 12 × 75 mm test tube. Then 0.6 mL of Ehrlich's reagent was added and color allowed to develop for 30 minutes. Absorbance was read at 565 nm against a reagent blank. Deionized water and 20 μg/mL hydroxyproline were used as the blank and standard, respectively.

### Serum oxidative stress evaluation

Eight rats from each group were randomly chosen for serum oxidative stress evaluation. During euthanasia, blood samples were collected and centrifuged at 1,400 g for 10 min. Aliquots of 0.5 mL serum were used to measure lipid hydroperoxide concentration, which was determined in medium containing methanol 90% (v/v), 250 μM ammonium ferrous sulfate, 100 μM xylenol orange, 25 mM sulfuric acid, and 4 mM butylated hydroxytoluene. The solution was incubated for 30 minutes at room temperature and measured at 560 nm [34].

Glutathione peroxidase was assayed in 15 μL of serum using 0.15 M phosphate buffer, pH 7.0, containing 5 mM EDTA, 0.1 mL of 0.0084 M NADPH, 4 μg of GSSG-reductase, 1.125 M sodium azide, and 0.15 M glutathione reduced form in a total volume of 0.3 mL [35].

Superoxide dismutase activity was determined In serum aliquots of 50 μL using its ability to inhibit reduction of nitroblue tetrazolium, in medium containing 50 mM phosphate buffer pH 7.4, 0.1 mM EDTA, 50 μM NBT, 78 μM NADH, and 3.3 μM phenazine methosulfate. One unit of superoxide dismutase was defined as the amount of protein needed to decrease the reference rate to 50% of maximum inhibition [36].

Enzyme activities were analyzed at 25°C using a microplate reader system (μQuant-MQX 200 with KC Junior software, Bio-TeK Instruments, Winooski, VT, USA). All reagents were purchased from Sigma (St. Louis, MO, USA).

### Real time RT-PCR (transcription polymerase chain reaction after reverse transcription) analysis

Ten rats from each group were randomly chosen for gene expression analysis. Total RNA was extracted from LV free wall with TRIzol Reagent (Invitrogen Life Technologies, Carlsbad, CA, USA) according to a previously described method [37,38]. Frozen muscles were mechanically homogenized on ice in 1 mL of ice-cold TRIzol reagent. Total RNA was solubilized in RNase-free H_2_O, incubated in DNase I (Invitrogen Life Technologies) to remove any DNA in the sample, and quantified by measuring optical density at 260 nm. RNA purity was ensured by obtaining a 260 / 280 nm optical density ratio of approximately 2.0. One microgram of RNA was reverse transcribed using High Capacity cDNA Reverse Transcription Kit in a total volume of 20 μL, according to standard methods (Applied Biosystems, Foster City, CA, USA). Aliquots of 2.5 μL (10–100 ng) of cDNA were then submitted to real-time PCR reaction using 10 μL 2× TaqMan® Universal PCR Master Mix (Applied Biosystems) and 1 μL of customized assay (20×) containing sense and antisense primers and TaqMan (Applied Biosystems) probe specific to each gene, sarcoplasmic reticulum calcium ATPase (SERCA) [TaqMan assay Rn00568762; Ref. seq. Genbank NM_017290], α-myosin heavy chain isoform (α-MyHC) [TaqMan assay Rn00568304_m1; Ref. seq. Genbank NM_017239.2], β-myosin heavy chain isoform (β-MyHC) [TaqMan assay Rn00568328_m1; Ref. seq. Genbank NM_017240.1], and atrial natriuretic peptide (ANP) [TaqMan assay Rn00561661_m1; Ref. seq. Genbank NM_012612.2]. Amplification and analysis were performed using Step One PlusTM Real Time PCR System (Applied Biosystems) according to manufacturer’s recommendation. As diabetes can modulate heart GAPDH content [39], expression data were normalized to cyclophilin expression [reference gene; TaqMan assay Rn00690933_m1; Ref. seq. Genbank NM_017101]. Reactions were performed in triplicate and expression levels calculated using the CT comparative method (2^-ΔΔCT^).

### Statistical analysis

Comparisons between groups were performed by the unpaired Student’s *t* test (expressed as mean ± standard deviation) when data showed normal distribution. When data showed non-normal distribution, comparisons between groups were made with the Mann-Whitney test (expressed as median and 25^th^ and 75^th^ percentiles).

## Results

During experiment, four SHR and six SHR-DM rats died. Three SHR-DM rats presented glycemia <220 mg/dL and were excluded from the study. At the end of the experiment, the diabetic group presented higher blood glucose and lower body weight than the control group. Systolic blood pressure was not significantly different between groups (Table 1).

**Table 1:** Glucose, body weight and systolic blood pressure

**Parameters**	**SHR (n = 21)**	**SHR-DM (n = 16)**	**P value**
Initial glucose (mg/dL)	95.1 ± 18.5	101 ± 8.88	0.461
Final glucose (mg/dL)	89.1 ± 21.1	487 ± 29	0.001
Initial BW (g)	370 ± 31	359 ± 22	0.118
Final BW (g)	339 ± 38	277 ± 26	0.001
Initial BP (mmHg)	203 ± 23	223 ± 43	0.139
Final BP (mmHg)	193 ± 34	201 ± 36	0.524

Data are expressed as mean ± standard deviation. SHR: spontaneously hypertensive rats without diabetes mellitus; SHR-DM: SHR with diabetes mellitus; BW: body weight; BP: systolic blood pressure. P value: result of statistical test comparing SHR-DM vs SHR (Student’s *t* test).

Table 2 shows cardiac structure data obtained at the end of the experiment. Heart rate was lower in the diabetic group. LV diastolic diameter normalized to body weight, LV systolic diameter, and left atrial diameter-to-body weight ratio were higher in the diabetic group. LV posterior wall, septal diastolic, and relative wall thickness were lower in SHR-DM. Tibia length, LV diastolic diameter/tibia length ratio and left atrial diameter/tibia length ratio did not differ between groups. LV functional data are shown in Table 3. The parameters of systolic function endocardial fractional shortening, ejection fraction, and posterior wall shortening velocity were lower in the diabetic group. Diastolic function evaluation showed decreased mitral A-wave and increased E/A ratio and isovolumetric relaxation time (IVRT) in SHR-DM. Mitral E-wave and E-wave deceleration time (EDT) did not differ between groups.

**Table 2:** Echocardiographic structural data

**Parameters**	**SHR (n = 21)**	**SHR-DM (n = 16)**	**P value**
HR (bpm)	322 ± 40	287 ± 40	0.010
LVDD (mm)	8.56 ± 1.03	8.73 ± 0.77	0.572
LVDD/BW (mm/kg)	24.2 (22.1 – 26.0)	31.2 (25.3 – 34.0)	0.001
LVDD/T (mm/cm)	2.02 ± 0.24	2.09 ± 0.19	0.445
LVSD (mm)	4.71 ± 1.27	5.54 ± 1.19	0.043
PWT (mm)	1.83 (1.75 – 1.90)	1.72 (1.66 – 1.79)	0.031
SWT (mm)	1.86 (1.77 – 1.91)	1.74 (1.66 – 1.83)	0.023
AO (mm)	4.50 (4.45 – 4.85)	4.45 (4.30 – 4.90)	0.701
LA (mm)	7.46 ± 1.12	7.51 ± 1.27	0.900
LA/BW (mm/kg)	21.5 ± 3.72	26.3 ± 5.82	0.003
LA/T (mm/cm)	1.75 ± 0.22	1.82 ± 0.33	0.513
LV mass (g)	1.24 (1.05 – 1.39)	1.18 (1.05 – 1.41)	0.673
LV mass/BW (g/Kg)	3.35 (3.12 – 4.04)	4.16 (3.27 – 5.05)	0.088
RWT	0.44 ± 0.07	0.40 ± 0.05	0.046
Tibia length (cm)	4.15 (4.10-4.19)	4.20 (4.10-4.20)	0.213

Data are expressed as mean ± standard deviation or median and 25^th^ and 75^th^ percentile. SHR: spontaneously hypertensive rats without diabetes mellitus; SHR-DM: SHR with diabetes mellitus; BW: body weight; HR: heart rate; LVDD and LVSD: left ventricular (LV) diastolic and systolic diameter, respectively; PWT: LV posterior wall thickness; SWT: LV septal wall thickness; AO: aortic diameter; LA: left atrial diameter; LV mass: LV mass; RWT: relative wall thickness; T: tibia length. P value: result of statistical test (Student’s *t* or Mann-Whitney tests).

**Table 3:** Echocardiographic left ventricular functional data

**Parameters**	**SHR (n = 21)**	**SHR-DM (n = 16)**	**P value**
FS (%)	45.8 ± 9.35	37.1 ± 8.94	0.005
Ejection fraction	0.83 ± 0.08	0.74 ± 0.11	0.005
PWSV (mm/s)	30.9 ± 7.10	26.4 ± 5.94	0.039
E-wave (cm/s)	77.1 ± 25.2	92.9 ± 30.4	0.090
A-wave (cm/s)	72.2 ± 37.1	34.4 ± 20.5	0.001
E/A	0.75 (0.62 – 4.37)	1.41 (1.19 – 6.43)	0.014
EDT (ms)	38.1 ± 5.67	33.2 ± 7.09	0.132
IVRT (ms)	36.9 ± 7.60	43.4 ± 6.44	0.008

Data are expressed as mean ± standard deviation or median and 25^th^ and 75^th^ percentile. SHR: spontaneously hypertensive rats without diabetes mellitus; SHR-DM: SHR with diabetes mellitus; FS: endocardial fractional shortening; PWSV: posterior wall shortening velocity; E: early diastolic mitral inflow velocity; A: late diastolic mitral inflow velocity; EDT: E-wave deceleration time; IVRT: isovolumetric relaxation time. P value: result of statistical test (Student’s *t* or Mann-Whitney tests).

**Figure 1:**
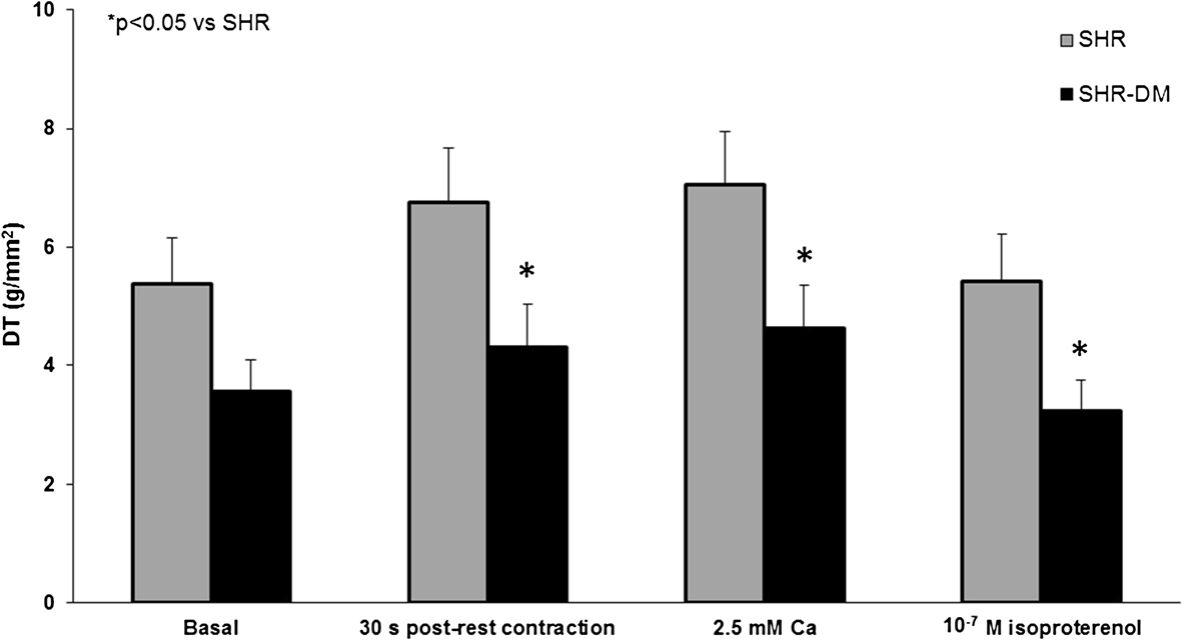
**Peak of developed tension (DT) during basal condition and after positive inotropic stimulation with 30 s post-rest contraction, 2.5 mM extracellular Ca**^**2+ **^**concentration, and 10**^**-7**^ **M isoproterenol added to nutrient solution.**

Papillary muscle functional data at basal condition with extracellular calcium concentration of 1.25 mM showed contractile impairment in SHR-DM characterized by prolonged time to peak tension and decreased maximum tension development rate. Peak developed tension, resting tension, and maximum tension decline rate did not differ between groups (Table 4). After positive inotropic stimulation with 30 s post-rest contraction, increased extracellular Ca^2+^ concentration (2.5 mM), and addition of 10^-7^ M isoproterenol to the nutrient solution, peak developed tension was lower in SHR-DM (Figure 1). After the three inotropic interventions, maximum tension development rate was smaller in SHR-DM, and maximum tension decline rate and resting tension did not differ between groups.

**Table 4:** Basal isolated papillary muscle data

**Parameters**	**SHR (n = 13)**	**SHR-DM (n = 12)**	**P value**
DT (g/mm^2^)	5.60 (2.57 – 7.99)	3.05 (2.17 – 5.42)	0.121
RT (g/mm^2^)	0.92 ± 0.37	0.81 ± 0.27	0.383
TPT (ms)	236 ± 30	264 ± 24	0.024
+dT/dt (g/mm²/s)	43.2 ± 22.5	25.7 ± 15.4	0.033
-dT/dt (g/mm²/s)	19.5 ± 9.54	15.3 ± 6.96	0.229
CSA (mm^2^)	1.04 ± 0.38	1.13 ± 0.30	0.511

Data are expressed as mean ± standard deviation or median and 25^th^ and 75^th^ percentile. SHR: spontaneously hypertensive rats without diabetes mellitus; SHR-DM: SHR with diabetes mellitus; DT: peak developed tension; RT: resting tension; TPT: time to peak tension; +dT/dt: maximum tension development rate; -dT/dt: maximum tension decline rate; CSA: papillary muscle cross sectional area. P value: result of statistical test (Student’s *t* or Mann-Whitney tests).

There was no significant difference between groups for LV hydroxyproline concentration (SHR 6.68 ± 1:52; SHR-DM 7.27 ± 2.28 mg/g; p = 0.50), interstitial collagen fraction (SHR 9.00 ± 2.90; SHR-DM 13.3 ± 7.60%; p = 0.11), and myocyte diameter [SHR 19.1 (17.0-22.4); SHR-DM 20.6 (19.6-21.0) μm; p = 0.62].

Serum oxidative stress parameters are presented in Table 5. Lipid hydroperoxide concentration was higher and superoxide dismutase and glutathione peroxidase were lower in SHR-DM.

**Table 5:** Evaluation of oxidative stress

**Variables**	**SHR (n = 8)**	**SHR-DM (n = 8)**	**P value**
Lipid hydroperoxide (nmol/ml)	8.80 ± 0.84	11.1 ± 1.04	0.001
Superoxide dismutase (U/mg)	687 ± 84	396 ± 50	0.001
Glutathione peroxidase (U/ml)	20.7 ± 1.98	17.6 ± 1.95	0.008

Data are expressed as mean ± standard deviation. SHR: spontaneously hypertensive rats without diabetes mellitus; SHR-DM: SHR with diabetes mellitus. P value: result of statistical test (Student’s t test).

Gene expression of SERCA, α-MyHC, and β-MyHC was not different between groups. The β-MyHC/α-MyHC ratio and ANP gene expression were higher in SHR-DM (Table 6).

**Table 6:** Evaluation of mRNA by real time PCR

**Variables**	**SHR (n = 10)**	**SHR-DM (n = 10)**	**P value**
SERCA	1.00 ± 0.71	1.79 ± 1.22	0.125
α-MyHC	1.00 ± 0.60	0.72 ± 0.45	0.294
β-MyHC	0.87 (0.70 – 1.17)	1.11 (0.78 – 1.28)	0.470
β-MyHC/α-MyHC	1.20 ± 0.60	2.26 ± 0.94	0.018
ANP	1.00 ± 0.38	1.88 ± 0.69	0.009

Data are expressed as mean ± standard deviation or median and 25^th^ and 75^th^ percentile (arbitrary units). SHR: spontaneously hypertensive rats without diabetes mellitus; SHR-DM: SHR with diabetes mellitus. SERCA: sarcoplasmic reticulum calcium ATPase; MyHC: myosin heavy chain; ANP: atrial natriuretic peptide; Cyclophilin: housekeeping gene used in standardizating target gene transcript product quantity. P value: result of statistical test (Student’s *t* or Mann-Whitney tests).

## Discussion

In this study we showed that streptozotocin-induced diabetes mellitus activates fetal gene program, increases serum oxidative stress, and impairs left ventricular and myocardial function in senescent spontaneously hypertensive rats.

To the best of our knowledge, this is the first study to evaluate the effects of streptozotocin-induced diabetes mellitus on the aged SHR heart. The spontaneously hypertensive rat is a well-established model of genetic hypertension [17-19]. SHR develops early arterial hypertension and left ventricular hypertrophy which evolve to cardiac failure usually between 18-22 months of age [18,40]. Taking into account the high prevalence of arterial hypertension and diabetes mellitus co-existing in the elderly, we induced diabetes at 18 months of age, when rats are considered senescent [18].

### Left ventricular and myocardial function

*In vivo* cardiac structures and systolic and diastolic function were analyzed by echocardiography. Diabetes mellitus increased LV systolic diameter without changing LV diastolic diameter and left atrium diameter in both absolute and values normalized to tibia length. When normalized to body weight, LV diastolic diameter and left atrium diameter were higher in SHR-DM than SHR. Normalization of cardiac structural parameters to body weight can lead to misinterpretation of results as cardiac chambers diameters are not exactly proportional to body weight [41]. Therefore, our data suggest that DM increases LV systolic diameter without dilating LV or left atrium in spontaneously hypertensive rats. LV posterior wall and septal diastolic thickness and LV relative wall thickness were lower in the diabetic group, while LV mass did not differ between groups. These results demonstrate that eccentric ventricular remodeling occurred in diabetic rats. SHR-DM also presented LV systolic function impairment, characterized by reduced endocardial fractional shortening, ejection fraction and posterior wall shortening velocity, and diastolic dysfunction characterized by reduced mitral A wave and increased E/A ratio and isovolumetric relaxation time. Eccentric ventricular remodeling and impaired LV systolic and diastolic function are commonly observed in diabetic cardiomyopathy in both clinical and experimental studies [2,4,42].

In this study, diabetes mellitus did not modulate blood pressure, which in accordance with Wienen et al. [11] in diabetic young SHR. Thus, we can discard blood pressure changes participating in the cardiac alterations observed in our SHR-DM.

Several pathophysiological mechanisms may be involved in LV dilation and dysfunction during diabetes [43]. Impaired myocardial contractility is an important cause of cardiac dilation and dysfunction [44]. In fact we found that myocardial systolic function, evaluated in isolated papillary muscle preparations, was depressed in diabetic rats, which presented increased time to peak tension and decreased + dT/dt. Contractile reserve was also impaired in SHR-DM as developed tension, unchanged at basal condition, was reduced after positive inotropic stimulation with post-rest contraction, increased extracellular Ca^2+^ concentration, and isoproterenol added to nutrient solution. Myocardial diastolic function was unchanged. Isolated papillary muscle preparations allow us to properly control preload and afterload and thus analyze intrinsic myocardial function. Therefore, we can conclude that depressed myocardial systolic function in combination with ventricular eccentric remodeling is involved in diabetes-induced LV functional impairment in aged SHR. Myocardial dysfunction has also been reported in adult diabetic hypertensive and normotensive rats [16,45].

In diabetes, several myocardial changes can be involved in myocardial dysfunction such as oxidative stress, myocardial fibrosis, fetal gene program activation, myocyte hypertrophy, and extracellular matrix metalloproteinases changes [13,46].

### Oxidative stress and fetal gene expression

Cardiac remodeling induced by diabetes and hypertension has been associated with increased oxidative stress [45,47]. In this study, we found increased lipid hydroperoxide concentration, which indicates increased serum free radicals, and a decrease in superoxide dismutase and glutathione peroxidase activity in SHR-DM. Therefore, diabetes mellitus increases myocardial oxidative stress in hypertensive rats. Although a causative role could not be demonstrated in this study, myocardial oxidative stress may have been involved in impaired myocardial and ventricular function in our SHR-DM. Studies have shown that increased oxidative stress jeopardizes myocardial function through mechanisms such as micro-vascular damage, abnormalities in calcium homeostasis, and endothelial dysfunction [8].

In this study, we observed a significant increase in atrial natriuretic peptide (ANP) gene expression in SHR-DM. Increased ANP gene expression has already been reported in diabetic rats [48]. In our study, increased ANP can be explained by the LV diastolic dysfunction with consequent LV diastolic pressure increase and ANP synthesis. There was no significant difference in SERCA, and α- and β-myosin gene expression. However, β/α-myosin gene expression ratio was higher in the diabetic group. This is consistent with the notion that alterations in glucose metabolism play a role in myosin heavy chain isoform changes [49]. In fact, Connelly et al. [50] found reduced α-myosin heavy chain and increased β-myosin heavy chain and β/α-myosin heavy chain ratio in diabetic rat hearts. Therefore, diabetes mellitus in SHR induces gene expression changes toward fetal gene program. In this study, the increased β/α-myosin ratio may have been responsible for the increased time to peak tension observed in SHR-DM papillary muscle.

In diabetes as well as in hypertension, the most prominent myocardial histopathological feature is fibrosis, which can be perivascular, interstitial, or both [8,51]. In this study, myocardial collagen content evaluated by myocardial hydroxyproline concentration and morfometric analysis in picrosirius red stained slides showed no significant difference between groups. These results are consistent with studies on the effects of combined hypertension and DM in mice [10] and SHR [52]. It should be pointed out that, although we found no significant differences in collagen content between SHR and SHR-DM, both groups presented increased myocardial fibrosis against literature data from normotensive rats [18,53,54]. Therefore, we can conclude that additional fibrosis is not involved in diabetes-induced impaired cardiac function in SHR.

Myocyte diameter was not different between groups. Myocyte hypertrophy is a common finding in cardiomyopathy induced by type 2 diabetes. In streptozotocin-induced diabetes, probably from hypoinsulinemia, myocardial hypertrophy has not commonly been observed [55].

One limitation of this study is that streptozotocin-induced type 1 DM is not the most common clinical situation in hypertensive aged patients. Type 1 and type 2 DM can differentially affect the heart as decreased systolic performance and delayed relaxation is more evident in type 1 DM and increased diastolic stiffness is remarkable in type 2 DM [56]. Therefore, additional studies are necessary to confirm our results in hypertensive rats with type 2 DM. Another limitation is the lack of LV end-diastolic pressure measurement, which is considered the gold standard for cardiac functional characterization [57]. Finally, the lower heart rate in SHR-DM (11% lower than in SHR) could have influenced left cardiac chamber sizes and LV function. However, isolated papillary muscle experiments, which allow myocardial function evaluation independent of cardiac load and heart rate, showed that systolic myocardial function was impaired in SHR-DM, confirming echocardiographic results.

## Conclusion

In conclusion, this study shows the adverse effects of type 1 diabetes when combined with genetic hypertension on cardiac remodeling in aged rats. Diabetes mellitus induces left atrium and ventricle dilation, left ventricular eccentric remodeling, and left ventricular and myocardial functional impairment. Cardiac remodeling is associated with increased oxidative stress and activated fetal gene program.

## Abbreviations

ANP: Atrial natriuretic peptide; AO: Aortic diameter; A-wave: Late diastolic mitral inflow velocity; AWT: Left ventricular diastolic anterior wall thickness; BP: Systolic blood pressure; BW: Body weight; CSA: Papillary muscle cross sectional area; DM: Diabetes mellitus; DT: Peak developed tension; -dT/dt: Maximum tension decline rate; +dT/dt: Maximum tension development rate; EDT: E-wave deceleration time; EF: Ejection fraction; E-wave: Early diastolic mitral inflow velocity; FS: Endocardial fractional shortening; HR: Heart rate; IVRT: Isovolumetric relaxation time; LA: Left atrium diameter; Lmax: Length-tension curve; LV: Left ventricular; LVDD: Left ventricular diastolic diameter; LV mass: Left ventricular mass; LVSD: Left ventricular systolic diameter; LVW: Left ventricular weight; MyHC: Myosin heavy chain; PWSV: Posterior wall shortening velocity; PWT: Left ventricular diastolic posterior wall thickness; RT: Resting tension; RT-PCR: Transcription polymerase chain reaction after reverse transcription; RWT: Relative wall thickness; SERCA: Sarcoplasmic reticulum calcium ATPase; SHR: Spontaneously hypertensive rats; SWT: Left ventricular septal wall thickness; T: Tibia length; TPT: Time to peak tension.

## Competing interest

The authors declare that they have no competing interests.

## Authors’ contributions

CMR and KO contributed to conception and design of study, acquisition of data, analysis and interpretation of data, and manuscript writing; NPX, DHSC, MDMC, CG, and RG contributed to data collection; AAHF, PFM, and ACC contributed to data collection and analysis; MPO contributed to manuscript writing. All authors have given final approval of the version to be published.
